# Supplementary material for: Proteomic Analysis of Domestic Cat Blastocysts and Their Secretome Produced in an In Vitro Culture System without the Presence of the Zona Pellucida
Source: Int J Mol Sci. 2024 Apr 14;25(8):4343. doi: 10.3390/ijms25084343 (PMC11050229; doi:10.3390/ijms25084343)
Supplement: Supplementary file 1 [file ijms-25-04343-s001.zip › ijms-2954893-supplementary.pdf]

Proteomic Analysis of Domestic Cat Blastocysts and Their Secretome Produced in an In Vitro Culture System without the Presence of the Zona Pellucida

Daniel Veraguas-Dávila [1,2,*], Camila Zapata-Rojas [2], Constanza Aguilera [3], Darling Saéz-Ruiz [2], Fernando Saravia [2], Fidel Ovidio Castro [2] and Lleretny Rodriguez-Alvarez [2]

[1] Escuela de Medicina Veterinaria, Departamento de Ciencias Agrarias, Facultad de Ciencias Agrarias y Forestales, Universidad Católica del Maule, Km 6 Los Niches, Curicó 3340000, Chile

[2] Department of Animal Science, Faculty of Veterinary Sciences, Universidad de Concepción, Av. Vicente Méndez 595, Chillan 3780000, Chile; mv.camilazapatarojas@gmail.com (C.Z.-R.); darlin.saez.r@gmail.com (D.S.-R.); fsaravia@udec.cl (F.S.); fidcastro@udec.cl (F.O.C.); llrodriguez@udec.cl (L.R.-A.)

[3] School of Veterinary Medicine, Faculty of Natural Sciences, San Sebastián University, Concepción 4081339, Chile; constanza.aguilera@uss.cl

* Correspondence: dveraguas@ucm.cl or daniveraguasdv@gmail.com

Supplementary files

S1. Principal component analysis (PCA) 2

S2. Quantifiable proteins of ZF and ZI blastocysts 3-8

S3. Detailed functional annotation of DEPs of blastocysts 9-11

S3.1-6. GO of proteins of ZF blastocysts (S3.1, S3.2, S3.3) and ZI blastocysts (S3.4, S3.5, S3.6) 12-17

S4. Proteins identified in the culture medium of blastocysts from the ZF and ZI groups…18-19

S5. GO annotation of the proteins identified in the culture 20-23

S6. Detailed functional annotation of proteins identified in conditioned medium 24-26

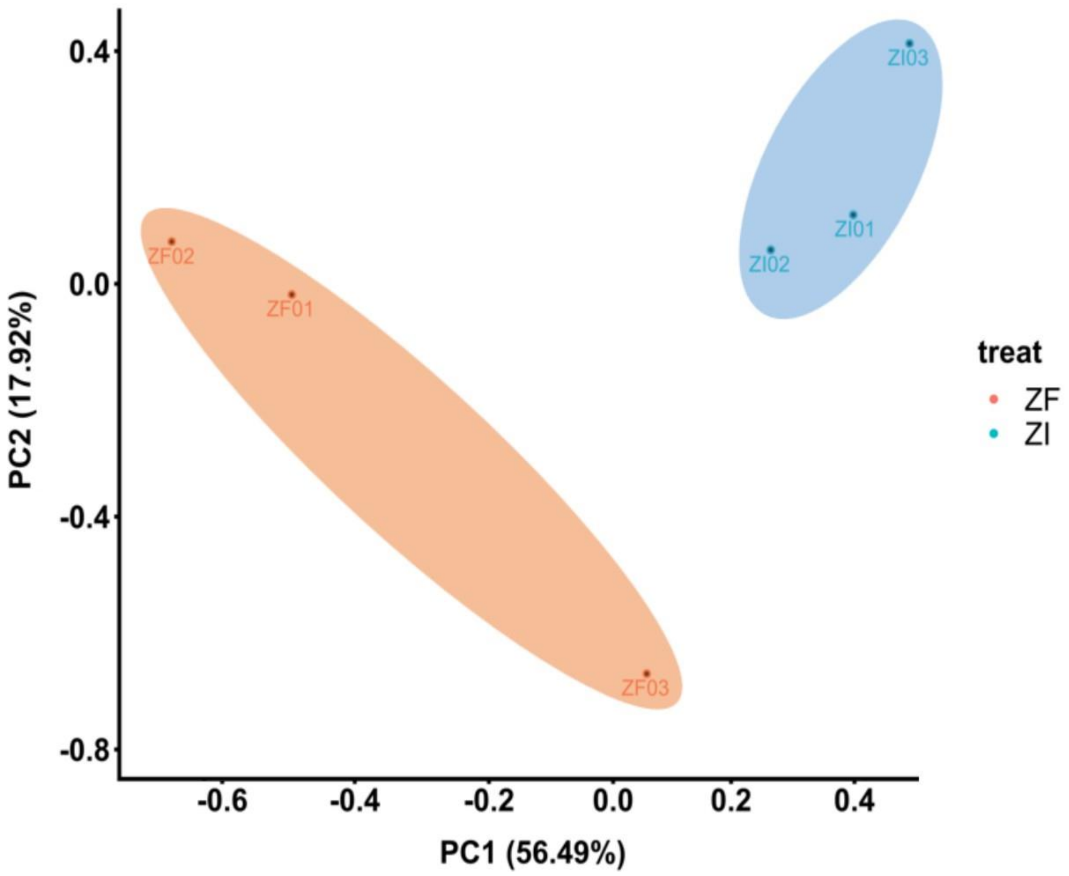

Figure S1. Principal component analysis (PCA). PCA of the total proteins detected in each replicate from the ZF (ZF01, ZF02 and ZF03) and ZI (ZI01, ZI02 and ZI03). The PCA shows clusters of expressed proteins that differ across the samples.

Table S2. Quantifiable proteins of ZF and ZI blastocysts. Quantifiable proteins identified in domestic cat blastocysts from the ZF and ZI groups. Differentially expressed proteins (DEPs) P < 0.05.

	Protein (ID)	Log2FC	P. Value
1	M3VZQ3|M3VZQ3_FELCA	3.17734479	8.8332E-05
2	A0A337SVF6|A0A337SVF6_FELCA	-2.81974135	0.00076552
3	A0A2I2UXE2|A0A2I2UXE2_FELCA	-2.65692623	0.00244662
4	A0A2I2U1C5|A0A2I2U1C5_FELCA	3.98060042	0.00311181
5	M3WCZ9|M3WCZ9_FELCA	-2.25459988	0.00357124
6	A0A5F5XCJ6|A0A5F5XCJ6_FELCA	-2.11846902	0.00388714
7	M3WHX6|M3WHX6_FELCA	-1.74681032	0.00401573
8	A0A337SCY3|A0A337SCY3_FELCA	-1.9626275	0.00586399
9	A0A2I2UZM2|A0A2I2UZM2_FELCA	1.95964111	0.00625748
10	A0A2I2U221|A0A2I2U221_FELCA	2.23313259	0.00612021
11	A0A337SW00|A0A337SW00_FELCA	-2.0509994	0.00760704
12	A0A2I2UXE4|A0A2I2UXE4_FELCA	2.31927503	0.00962896
13	M3VYI7|M3VYI7_FELCA	2.71239201	0.00899639
14	M3VX46|M3VX46_FELCA	1.61502009	0.01107067
15	A0A337S6R5|A0A337S6R5_FELCA	2.42297848	0.01078582
16	M3W9Y8|M3W9Y8_FELCA	2.01100493	0.01179752
17	M3WAW0|M3WAW0_FELCA	-1.48787899	0.01293448
18	A0A5F5XWX3|A0A5F5XWX3_FELCA	-1.82092779	0.01365135
19	A0A5F5XQQ6|A0A5F5XQQ6_FELCA	-1.9562502	0.01636457
20	M3WCX7|M3WCX7_FELCA	1.87232765	0.01809163
21	A0A5F5Y0Y9|A0A5F5Y0Y9_FELCA	1.89221244	0.01811358
22	A0A2I2UL05|A0A2I2UL05_FELCA	-1.5655253	0.01909538
23	M3VW27|M3VW27_FELCA	-1.46532	0.01962583
24	M3WD17|M3WD17_FELCA	-1.36580332	0.0206993
25	A0A337SJJ9|A0A337SJJ9_FELCA	1.42487437	0.02292737
26	M3W1X7|M3W1X7_FELCA	-1.60814943	0.02304837
27	A0A5F5XWD4|A0A5F5XWD4_FELCA	2.23313382	0.02424332
28	M3W9X1|M3W9X1_FELCA	3.21413703	0.02400918
29	A0A2I2UMD6|A0A2I2UMD6_FELCA	1.48842654	0.02362781
30	M3X4Y3|M3X4Y3_FELCA	-1.32858383	0.02750559
31	M3WFT4|M3WFT4_FELCA	1.34473579	0.02860092
32	M3WDF5|M3WDF5_FELCA	-1.44449596	0.03319027
33	Q66RN5|EF1A1_FELCA	1.51020066	0.03705747
34	M3WVC7|M3WVC7_FELCA	1.35282905	0.04453224
35	A0A5F5XYM1|A0A5F5XYM1_FELCA	-2.29930597	0.03963366
36	A0A2I2U7Z5|A0A2I2U7Z5_FELCA	1.60935135	0.0440415
37	M3WIA4|M3WIA4_FELCA	-1.28571479	0.04705984
38	A0A2I2V068|A0A2I2V068_FELCA	-1.35619626	0.04892405
39	M3WJL8|M3WJL8_FELCA	1.29586264	0.04988445
40	M3WRT2|M3WRT2_FELCA	2.0962291	0.04569637

41	M3W9D5|M3W9D5_FELCA	-1.20664542	0.04717624
42	A0A337S416|A0A337S416_FELCA	1.3425531	0.04971628
43	M3WJZ7|M3WJZ7_FELCA	-1.28876733	0.05618766
44	M3W6H0|M3W6H0_FELCA	-1.51076432	0.05877985
45	A0A2I2U4B2|A0A2I2U4B2_FELCA	-1.21562436	0.06292421
46	A0A5F5Y5E1|A0A5F5Y5E1_FELCA	-1.94225307	0.06737603
47	M3X1T7|M3X1T7_FELCA	2.8291405	0.07447896
48	A0A5F5XQT3|A0A5F5XQT3_FELCA	-1.03582209	0.06176732
49	M3W6X3|M3W6X3_FELCA	1.11369034	0.06106935
50	M3WXV4|M3WXV4_FELCA	1.14585085	0.07820449
51	M3WRZ7|M3WRZ7_FELCA	1.19497296	0.07464491
52	A0A5F5XFM1|A0A5F5XFM1_FELCA	-1.32299395	0.08053958
53	A0A2I2UNG3|A0A2I2UNG3_FELCA	1.76317592	0.07434401
54	M3XDX6|M3XDX6_FELCA	-1.11660959	0.07863656
55	A0A337SQQ5|A0A337SQQ5_FELCA	-1.30598621	0.0871821
56	A0A2I2UD00|A0A2I2UD00_FELCA	-1.18176787	0.08979881
57	A0A2I2UUV9|A0A2I2UUV9_FELCA	1.09493502	0.09261486
58	A0A5F5XM88|A0A5F5XM88_FELCA	-1.33422208	0.08927905
59	A0A5F5XNM3|A0A5F5XNM3_FELCA	-0.94863444	0.07606561
60	M3WUL6|M3WUL6_FELCA	1.24481136	0.09206694
61	A0A337RZN8|A0A337RZN8_FELCA	1.01758194	0.09952202
62	A0A2I2U5M9|A0A2I2U5M9_FELCA	1.01501299	0.10796034
63	A0A2I2UQ30|A0A2I2UQ30_FELCA	-1.10497045	0.1026632
64	M3W6N1|M3W6N1_FELCA	-0.87423562	0.09685437
65	M3WRM0|M3WRM0_FELCA	-0.95478767	0.12936561
66	M3W103|M3W103_FELCA	-1.1497569	0.12663303
67	A0A337SKJ9|A0A337SKJ9_FELCA	-0.88254023	0.1291582
68	M3XAK5|M3XAK5_FELCA	-0.93351261	0.12121576
69	M3VVI7|M3VVI7_FELCA	0.88137742	0.12709696
70	A0A337SU81|A0A337SU81_FELCA	-2.42330235	0.12053453
71	M3WQJ0|M3WQJ0_FELCA	-0.955751	0.11180913
72	A0A2I2UIR6|A0A2I2UIR6_FELCA	-1.43879033	0.13210278
73	A0A337SGK3|A0A337SGK3_FELCA	-0.97457468	0.13432965
74	A0A337S2X4|A0A337S2X4_FELCA	1.45180618	0.12727104
75	M3VWC7|M3VWC7_FELCA	-2.8404019	0.11667173
76	A0A2I2V1C1|A0A2I2V1C1_FELCA	-2.50453648	0.13117932
77	M3XE20|M3XE20_FELCA	1.18236665	0.13884542
78	M3VW05|M3VW05_FELCA	0.87555343	0.15495318
79	A0A337SDT4|A0A337SDT4_FELCA	-0.94151244	0.14172712
80	M3W340|M3W340_FELCA	1.00393666	0.15457413
81	M3WWP2|M3WWP2_FELCA	1.0519005	0.1552471
82	M3W1E8|M3W1E8_FELCA	-0.71424771	0.13951312
83	M3WMR1|M3WMR1_FELCA	-0.98450477	0.14937222
84	A0A337SJM7|A0A337SJM7_FELCA	1.430515	0.16597559
85	D3U660|D3U660_FELCA	-0.89811593	0.17749818

86	A0A337RXE8|A0A337RXE8_FELCA	1.39230122	0.17120384
87	M3WFR2|M3WFR2_FELCA	0.74858779	0.16297069
88	A0A5F5Y0E9|A0A5F5Y0E9_FELCA	0.87421188	0.16993118
89	M3W8D3|M3W8D3_FELCA	-1.11652385	0.19614802
90	M3W6N3|M3W6N3_FELCA	-0.84229759	0.20542554
91	A0A337STW1|A0A337STW1_FELCA	-1.18300428	0.19807428
92	A0A337SK34|A0A337SK34_FELCA	-0.71507341	0.1977503
93	A0A337SEI8|A0A337SEI8_FELCA	0.83705413	0.187847
94	A0A5F5XJ28|A0A5F5XJ28_FELCA	1.0281029	0.20696765
95	M3XCN6|M3XCN6_FELCA	0.74630243	0.18057895
96	M3VVN9|M3VVN9_FELCA	-1.91235804	0.18946062
97	M3W5B5|M3W5B5_FELCA	1.15469796	0.19471384
98	M3WIJ6|M3WIJ6_FELCA	0.85896043	0.19457348
99	M3WZB0|M3WZB0_FELCA	0.79449334	0.20071081
100	M3WIY9|M3WIY9_FELCA	0.68731582	0.21730774
101	A0A2I2U4U9|A0A2I2U4U9_FELCA	-0.6948812	0.23504809
102	M3XAB2|M3XAB2_FELCA	-0.63181561	0.21039822
103	M3WEG0|M3WEG0_FELCA	0.73614418	0.21328651
104	A0A337S1C3|A0A337S1C3_FELCA	-0.61691418	0.19212901
105	A0A337S574|A0A337S574_FELCA	-0.87744834	0.22741398
106	A0A2I2U5B1|A0A2I2U5B1_FELCA	0.6893842	0.23889908
107	M3WES0|M3WES0_FELCA	1.33559327	0.24972994
108	A0A5F5XZJ4|A0A5F5XZJ4_FELCA	0.68525792	0.27332331
109	A0A2I2V4V0|A0A2I2V4V0_FELCA	-1.22412716	0.25283299
110	M3W0Z9|M3W0Z9_FELCA	1.25556378	0.25675866
111	M3WXZ4|M3WXZ4_FELCA	0.74101269	0.23774364
112	M3W0S1|M3W0S1_FELCA	-0.74563497	0.18870746
113	M3W314|M3W314_FELCA	0.63825065	0.25602845
114	M3W616|M3W616_FELCA	-1.61546534	0.24485034
115	M3WFE1|M3WFE1_FELCA	0.84877822	0.19648222
116	M3W6I2|M3W6I2_FELCA	-0.7070366	0.24716385
117	M3XFH0|M3XFH0_FELCA	0.89905949	0.25148967
118	M3WG78|M3WG78_FELCA	0.69794975	0.26145728
119	A0A5F5XXV6|A0A5F5XXV6_FELCA	-0.72392569	0.30454032
120	A0A337S6S4|A0A337S6S4_FELCA	0.60932632	0.26272722
121	A0A2I2V257|A0A2I2V257_FELCA	0.65866376	0.26273789
122	A0A5F5XLI3|A0A5F5XLI3_FELCA	-1.20921034	0.30052652
123	M3X673|M3X673_FELCA	1.29524112	0.29625437
124	A0A0A0MPX9|A0A0A0MPX9_FELCA	0.56673339	0.2935787
125	A0A2I2UFN5|A0A2I2UFN5_FELCA	0.5739124	0.345949
126	A0A5F5XTM3|A0A5F5XTM3_FELCA	-0.77298411	0.30682978
127	M3VWM9|M3VWM9_FELCA	-0.73283126	0.37583264
128	A0A5F5XI95|A0A5F5XI95_FELCA	-0.69785452	0.33005281
129	M3VX06|M3VX06_FELCA	-0.63290582	0.40177814
130	A0A337S0E1|A0A337S0E1_FELCA	-1.59264676	0.35190383

131	M3W8Q7|M3W8Q7_FELCA	0.54243869	0.40418621
132	M3X7Z9|M3X7Z9_FELCA	0.63226259	0.40663471
133	A0A337SBS4|A0A337SBS4_FELCA	0.61730868	0.36518077
134	A0A5F5XDM3|A0A5F5XDM3_FELCA	-1.04796814	0.38457297
135	A0A2I2V2E9|A0A2I2V2E9_FELCA	-0.54045608	0.37838812
136	M3WN96|M3WN96_FELCA	-0.97199818	0.39857618
137	A0A337S5W7|A0A337S5W7_FELCA	-0.6470899	0.44761128
138	A0A337SKI4|A0A337SKI4_FELCA	0.79674309	0.42717936
139	A0A2I2V256|A0A2I2V256_FELCA	-1.21278287	0.41387961
140	A0A5F5XE26|A0A5F5XE26_FELCA	0.72716569	0.40729381
141	M3WM64|M3WM64_FELCA	0.78744828	0.44932791
142	A0A5F5XVC1|A0A5F5XVC1_FELCA	-0.74508586	0.45003252
143	A0A2I2UP60|A0A2I2UP60_FELCA	0.48528117	0.39241787
144	M3VY74|M3VY74_FELCA	-0.51296096	0.48588117
145	M3W9U6|M3W9U6_FELCA	-1.00312623	0.46131722
146	A0A2I2UG69|A0A2I2UG69_FELCA	0.66929167	0.49381499
147	M3VY56|M3VY56_FELCA	-0.53077197	0.51477886
148	A0A2I2UNB8|A0A2I2UNB8_FELCA	-0.39333699	0.44012135
149	A0A337SAA8|A0A337SAA8_FELCA	-1.15669399	0.4616709
150	M3VWY7|M3VWY7_FELCA	0.54230663	0.41443626
151	M3WNV1|M3WNV1_FELCA	0.6966889	0.49031456
152	A0A2I2UJE2|A0A2I2UJE2_FELCA	0.66064069	0.4133928
153	A0A5F5XQH4|A0A5F5XQH4_FELCA	0.5550161	0.43508366
154	M3WGE6|M3WGE6_FELCA	0.74753285	0.41061425
155	M3X2Q9|M3X2Q9_FELCA	-0.4897043	0.48225859
156	A0A2I2U902|A0A2I2U902_FELCA	0.73084154	0.53015201
157	A0A2I2UDQ5|A0A2I2UDQ5_FELCA	0.55171635	0.49419813
158	M3W7N2|M3W7N2_FELCA	0.47614104	0.415903
159	A0A5F5XE84|A0A5F5XE84_FELCA	0.5693967	0.43053054
160	M3VV33|M3VV33_FELCA	-0.43455161	0.42186376
161	A0A2I2UHR0|A0A2I2UHR0_FELCA	0.55252544	0.50489897
162	A0A2I2V2I3|A0A2I2V2I3_FELCA	-0.84502585	0.55533422
163	M3WSM7|M3WSM7_FELCA	0.55817512	0.47007981
164	M3VX55|M3VX55_FELCA	0.4155886	0.47936794
165	M3XCQ1|M3XCQ1_FELCA	-0.46820336	0.47874947
166	A0A337SFA0|A0A337SFA0_FELCA	0.5220583	0.50596542
167	M3WU44|M3WU44_FELCA	-0.5840467	0.40386813
168	A0A337S2D3|A0A337S2D3_FELCA	0.27982289	0.65995173
169	A0A337S266|A0A337S266_FELCA	-0.43491997	0.59816099
170	P63052|RL40_FELCA	0.40760661	0.45350201
171	M3VZE2|M3VZE2_FELCA	0.45272905	0.53028123
172	M3VYQ2|M3VYQ2_FELCA	0.34418969	0.48789018
173	A0A5F5XEX8|A0A5F5XEX8_FELCA	-0.42198273	0.56543779
174	M3W484|M3W484_FELCA	-0.31737862	0.71123519
175	M3WAQ4|M3WAQ4_FELCA	0.41428386	0.59670788

176	M3X6Y9|M3X6Y9_FELCA	-0.5272906	0.70475752
177	A0A2I2U0U0|A0A2I2U0U0_FELCA	0.49135937	0.66422395
178	A0A2I2UD94|A0A2I2UD94_FELCA	-0.27499601	0.74736059
179	M3X8C2|M3X8C2_FELCA	0.27777717	0.68644143
180	M3WFX6|M3WFX6_FELCA	0.34794819	0.70357863
181	A0A2I2U4W2|A0A2I2U4W2_FELCA	0.46827761	0.50778625
182	M3WGK3|M3WGK3_FELCA	-0.35853682	0.49388268
183	M3XB84|M3XB84_FELCA	-0.54178942	0.5353923
184	M3WLH5|M3WLH5_FELCA	-0.15826925	0.81602143
185	A0A337S2E2|A0A337S2E2_FELCA	-0.23048676	0.71707321
186	A0A337SAT1|A0A337SAT1_FELCA	-0.40752042	0.54197182
187	M3WAB5|M3WAB5_FELCA	0.14051258	0.84858983
188	M3VXZ1|M3VXZ1_FELCA	0.30650203	0.66882377
189	A0A5F5XKD4|A0A5F5XKD4_FELCA	0.16216635	0.78687204
190	P61246|RS3A_FELCA	-0.42039794	0.54178873
191	M3X3M7|M3X3M7_FELCA	-0.4027443	0.82418817
192	M3VXE9|M3VXE9_FELCA	0.48421677	0.5614932
193	M3VVZ2|M3VVZ2_FELCA	0.07473626	0.94205655
194	A0A2I2UFE0|A0A2I2UFE0_FELCA	0.19756537	0.78368149
195	M3XCH0|M3XCH0_FELCA	0.33154207	0.69930009
196	M3WAZ1|M3WAZ1_FELCA	0.0688818	0.95978421
197	M3WY95|M3WY95_FELCA	0.34809153	0.61956759
198	M3X2J1|M3X2J1_FELCA	0.38207968	0.55067371
199	A0A2I2UWM9|A0A2I2UWM9_FELCA	0.05780446	0.9254989
200	A0A2I2U1M4|A0A2I2U1M4_FELCA	0.47114034	0.61481224
201	M3VUH3|M3VUH3_FELCA	-0.08414469	0.94342427
202	A0A5F5XKM9|A0A5F5XKM9_FELCA	-0.241069	0.65125065
203	A0A2I2URI6|A0A2I2URI6_FELCA	-0.60474723	0.69245125
204	M3W668|M3W668_FELCA	0.24647312	0.66201051
205	A0A2I2U5K7|A0A2I2U5K7_FELCA	-0.20079229	0.71490421
206	A0A2I2V460|A0A2I2V460_FELCA	-0.23749236	0.70739198
207	A0A2I2U8W9|A0A2I2U8W9_FELCA	-0.4689799	0.64817985
208	A0A2I2UXL1|A0A2I2UXL1_FELCA	-0.02707674	0.97024054
209	A0A2I2U9Q8|A0A2I2U9Q8_FELCA	-0.35372991	0.62093985
210	A0A2I2U9C6|A0A2I2U9C6_FELCA	0.43543891	0.68076518
211	M3W4G0|M3W4G0_FELCA	0.29203187	0.62429355
212	A0A2I2UY31|A0A2I2UY31_FELCA	0.24485541	0.6303822
213	M3VZT0|M3VZT0_FELCA	0.28066303	0.63175032
214	A0A337SGS6|A0A337SGS6_FELCA	-0.21434273	0.713302
215	A0A2I2V0F1|A0A2I2V0F1_FELCA	0.0269977	0.97111462
216	A0A5F5XE47|A0A5F5XE47_FELCA	-0.33766502	0.70028922
217	A0A2I2UJH0|A0A2I2UJH0_FELCA	0.32258437	0.67086999
218	A0A5F5XV94|A0A5F5XV94_FELCA	-0.29761638	0.60725388
219	M3W584|M3W584_FELCA	0.16394197	0.80233478
220	A0A337S3P4|A0A337S3P4_FELCA	0.19294274	0.69024359

221	M3WCU9|M3WCU9_FELCA	-0.22366223	0.75470213
222	M3WGN8|M3WGN8_FELCA	0.29436657	0.68817701
223	M3W9K1|M3W9K1_FELCA	-0.41375661	0.62547059
224	Q9N2D5|G3P_FELCA	0.28868361	0.71679951
225	A0A337SJV5|A0A337SJV5_FELCA	0.20340093	0.69208425
226	M3WFL8|M3WFL8_FELCA	0.26081364	0.6323484
227	A0A337S498|A0A337S498_FELCA	0.10694867	0.88774223
228	M3WWI9|M3WWI9_FELCA	0.00561339	0.9923296
229	A0A2I2UFB9|A0A2I2UFB9_FELCA	0.2004487	0.81835163
230	A0A2I2UTE6|A0A2I2UTE6_FELCA	-0.06685416	0.9407761
231	M3WZA4|M3WZA4_FELCA	0.36516127	0.78176555
232	M3W059|M3W059_FELCA	-0.08800975	0.90622452
233	M3W6M1|M3W6M1_FELCA	0.19046829	0.79644861
234	M3WEF9|M3WEF9_FELCA	0.04460123	0.93370804
235	A0A2I2V3D5|A0A2I2V3D5_FELCA	-0.04726234	0.95249919
236	A8WA76|A8WA76_FELCA	-0.00154657	0.99752947
237	K0J107|MDHM_FELCA	-0.09861651	0.84915846
238	A0A2I2U8U0|A0A2I2U8U0_FELCA	0.2415807	0.78211448
239	M3W5T1|M3W5T1_FELCA	-0.10418937	0.87296297
240	M3XFZ1|M3XFZ1_FELCA	-0.04177231	0.93146945
241	Q66WF5|RL24_FELCA	-0.08975336	0.90495434
242	A0A5F5Y6Z4|A0A5F5Y6Z4_FELCA	-0.06822556	0.89754353
243	A0A2I2ULA7|A0A2I2ULA7_FELCA	-0.13665265	0.80711816
244	A0A337SMT8|A0A337SMT8_FELCA	0.09408336	0.91752904
245	M3VUG5|M3VUG5_FELCA	-0.14139884	0.87214441
246	M3WVY3|M3WVY3_FELCA	-0.02400945	0.95681854
247	M3X4F1|M3X4F1_FELCA	0.01954638	0.96919767
248	M3W064|M3W064_FELCA	0.07851178	0.89797205
249	M3WMZ2|M3WMZ2_FELCA	-0.18206107	0.70626683
250	M3XA07|M3XA07_FELCA	-0.00398996	0.99387616

Table S3. Detailed functional annotation of DEPs (Fig.6 Pie charts). Upregulated proteins: A) Molecular function,
B) Biological Process, and C) Cellular component. Downregulated proteins: D) Molecular function, E) Biological Process, F) Cellular component.

A) Upregulated proteins. Molecular function GO:0003674

1. Cytoskeletal motor activity GO:0003774 (MYH9).	1
2. Binding GO:0005488 (MYH9, TUFM, IGF2BP1, HNRNPH1, HSP90B1, CCT8, RPL8, RPL18, HSPA8, EZR, COX5B, EEF1A1, EEF1B2, HDLBP, RNPS1, YWHAZ, HSPA5).	17
3. Catalytic activity GO:0003824 (PDIA4, TUFM, ATP5PF, HSP90B1, CCT8, HSPA8, EEF1A1, EEF1B2, HSPA5, ALDOA).	10
4. Translation regulator activity GO:0045182 (EEF1A1).	1
5. Molecular adaptor activity GO:0060090 (IGF2BP1, TLE6).	2
6. Structural molecule activity GO:0005198 (RPL8, RPL18).	2
7. Molecular function regulator activity GO:0098772 (EEF1B2).	1
8. ATP-dependent activity GO:0140657 (HSP90B1, CCT8, HSPA8, HSPA5).	4
9. Molecular sequestering activity GO:0140313 (YWHAZ).	1
10. Transporter activity GO:0005215 (ATP5PF).	1
11. Transcription regulator activity GO:0140110 (TUFM, TLE6).	2
12. Protein folding chaperone GO:0044183 (HSP90B1, CCT8, HSPA8, HSPA5).	4
13. Unassigned (BCAP31).	1

B) Upregulated proteins. Biological process GO:0008150

1. Cellular process GO:0009987 (PDIA4, TUFM, IGF2BP1, ATP5PF, HSP90B1, CCT8, RPL8, RPL18, HSPA8, EEF1B2, COX5B, BCAP31, EEF1A1, RNPS1, YWHAZ, HSPA5, ALDOA).	17
2. Response to stimulus GO:0050896 (PDIA4, YWHAZ, HSPA5, IGF2BP1, HSP90B1, EEF1A1).	6
3. Metabolic process GO:0008152 (PDIA4, TUFM, IGF2BP1, ATP5PF, RPL8, RPL18, HSPA8, EEF1B2, COX5B, EEF1A1, RNPS1, HSPA5, ALDOA, HSP90B1, CCT8, YWHAZ).	16
4. Reproduction GO:0000003 (CCT8, TLE6).	2
5. Biological regulation GO:0065007 (IGF2BP1, YWHAZ, HSPA5, TLE6, HSP90B1, CCT8, EEF1A1).	7
6. Developmental process GO:0032502 (IGF2BP1, YWHAZ, HSPA5, TLE6).	4
7. Multicellular organismal process GO:0032501 (IGF2BP1, HSPA5).	2
8. Localization GO:0051179 (IGF2BP1, HSPA8, BCAP31, HSP90B1, CCT8, YWHAZ, HSPA5).	7
9. Unassigned (MYH9, HNRNPH1, EZR, HDLBP).	4

C) Upregulated proteins. Cellular component GO:0005575

1. Cytoskeleton GO:0005856 (MYH9, CCT8, EZR).	3
2. Extracellular region GO:0005576 (PDIA4).	1
3.	
4. Endoplasmic reticulum GO:0005783 (PDIA4, HSP90B1, RPL18, EEF1B2, BCAP31, HSPA5).	6
5. Organelle lumen GO:0043233 (PDIA4, IGF2BP1, HNRNPH1, HSP90B1, CCT8, EEF1A1, RNPS1, HSPA5).	8
6. Mitochondrion GO:0005739 (TUFM, ATP5PF, COX5B, HSPA5).	4
7. Nucleus GO:0005634 (IGF2BP1, HNRNPH1, CCT8, HSPA8, EEF1A1, HDLBP, RNPS1, HSPA5, TLE6).	9
8. Cytosol GO:0005829 (IGF2BP1, HSP90B1, CCT8, RPL8, RPL18, HSPA8, EEF1B2, YWHAZ, HSPA5).	9
9. Protein-containing complex GO:0032991 (IGF2BP1, ATP5PF, HSP90B1, CCT8, RPL8, RPL18, COX5B, RNPS1, HSPA5, TLE6).	10
10. Cell projection GO:0042995 (IGF2BP1, CCT8, HSPA8, EZR).	4
11. non-membrane-bounded organelle GO:0043228 (IGF2BP1, RPL8, RPL18, EEF1A1).	4
12. Organelle envelope GO:0031967 (ATP5PF, COX5B).	2
13. Cytoplasmic vesicle GO:0031410 (HSP90B1, HSPA5).	2
14. Vacuole GO:0005773 (HSPA8).	1
15. Plasma membrane GO:0005886 (HSPA8, EZR, EEF1A1, HSPA5).	4
16. Unassigned (ALDOA).	1

D) Downregulated proteins. Molecular function GO:0003674

1. Catalytic activity GO:0003824 (UQCRFS1, EMG1, PLK1).	3
2. Binding GO:0005488 (LGALS1, UQCRFS1, RPL12, TRA2B, RRP9, EMG1, PLK1, KHDC3L, H2BC3, H2BC18, GNG12, HMGA1).	12
3. Structural molecule activity GO:0005198 (RPL12, RPS25, RPL36A, COL1A2, H2BC3, H2BC18).	6
4. Antioxidant activity GO:0016209 (PRXL2A).	1
5. Molecular carrier activity GO:0140104 (KPNA2).	1
6. Transcription regulator activity GO:0140110 (HMGA1).	1
7. Transporter activity GO:0005215 (UQCRFS1).	1
8. Molecular adaptor activity GO:0060090 (HMGA1).	1
9. Unassigned (COA3, PSME3IP1, YIPF3).	4

E) Downregulated proteins. Biological process GO:0008150

1. Cellular process GO:0009987 (COA3, UQCRFS1, RPL12, TRA2B, RPS25, RRP9, PRXL2A, EMG1, PLK1, KPNA2, RPL36A, GNG12, HMGA1).	13
2. Biological regulation GO:0065007 (COA3, TRA2B, PLK1, GNG12, HMGA1).	5
3. Metabolic process GO:0008152 (COA3, UQCRFS1, RPL12, TRA2B, RPS25, RRP9, EMG1, PLK1, RPL36A, HMGA1).	10
4. Response to stimulus GO:0050896 (TRA2B, PRXL2A, PLK1, GNG12, HMGA1).	5
5. Homeostatic process GO:0042592 (TRA2B).	1
6. Developmental process GO:0032502 (TRA2B, EMG1).	2
7. Multicellular organismal process GO:0032501 (TRA2B, PLK1).	2
8. Localization GO:0051179 (PLK1, KPNA2).	2

9. Reproduction GO:0000003 (PLK1).	1
10. Unassigned (LGALS1, PSME3IP1, KHDC3L, H2BC3, YIPF3, H2BC18, COL1A2).	7

F) Downregulated proteins. Cellular component GO:0005575

1. Mitochondrion GO:0005739 (COA3, UQCRFS1).	2
2. Organelle envelope GO:0031967 (UQCRFS1, TRA2B).	2
3. Protein-containing complex GO:0032991 (UQCRFS1, TRA2B, RPS25, RRP9, EMG1, KHDC3L, RPL36A, H2BC3, H2BC18, GNG12).	10
4. Membrane GO:0016020 (PSME3IP1, YIPF3).	2
5. Cytosol GO:0005829 (RPL12, RPS25, H2BC3, H2BC18).	4
6. Ribosome GO:0005840 (RPL12).	1
7. Nucleus GO:0005634 (TRA2B, RRP9, EMG1, PLK1, H2BC3, H2BC18, HMGA1).	7
8. Organelle lumen GO:0043233 (TRA2B, RRP9, EMG1, H2BC3, H2BC18).	5
9. non-membrane-bounded organelle GO:0043228 (RPS25, RRP9, EMG1, RPL36A, H2BC3, H2BC18).	6
10. Cytoplasm GO:0005737 (PRXL2A).	1
11. Nuclear chromosome GO:0000228 (PLK1).	1
12. Chromosome GO:0005694 (PLK1, HMGA1).	2
13. Microtubule organizing center GO:0005815 (PLK1).	1
14. Cytoskeleton GO:0005856 (PLK1).	1
15. Golgi apparatus GO:0005794 (YIPF3).	1
16. Cytoplasmic vesicle GO:0031410 (YIPF3).	1
17. Plasma membrane GO:0005886 (GNG12).	1
18. Unassigned (LGALS1, KPNA2, COL1A2).	3

PANTHER GO-Slim Biological Process - ZF Blastocysts

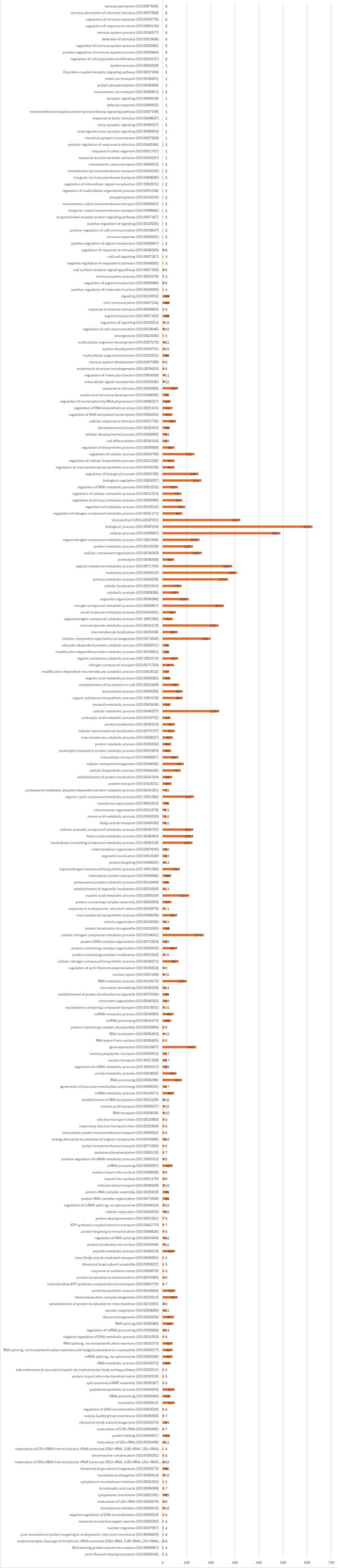

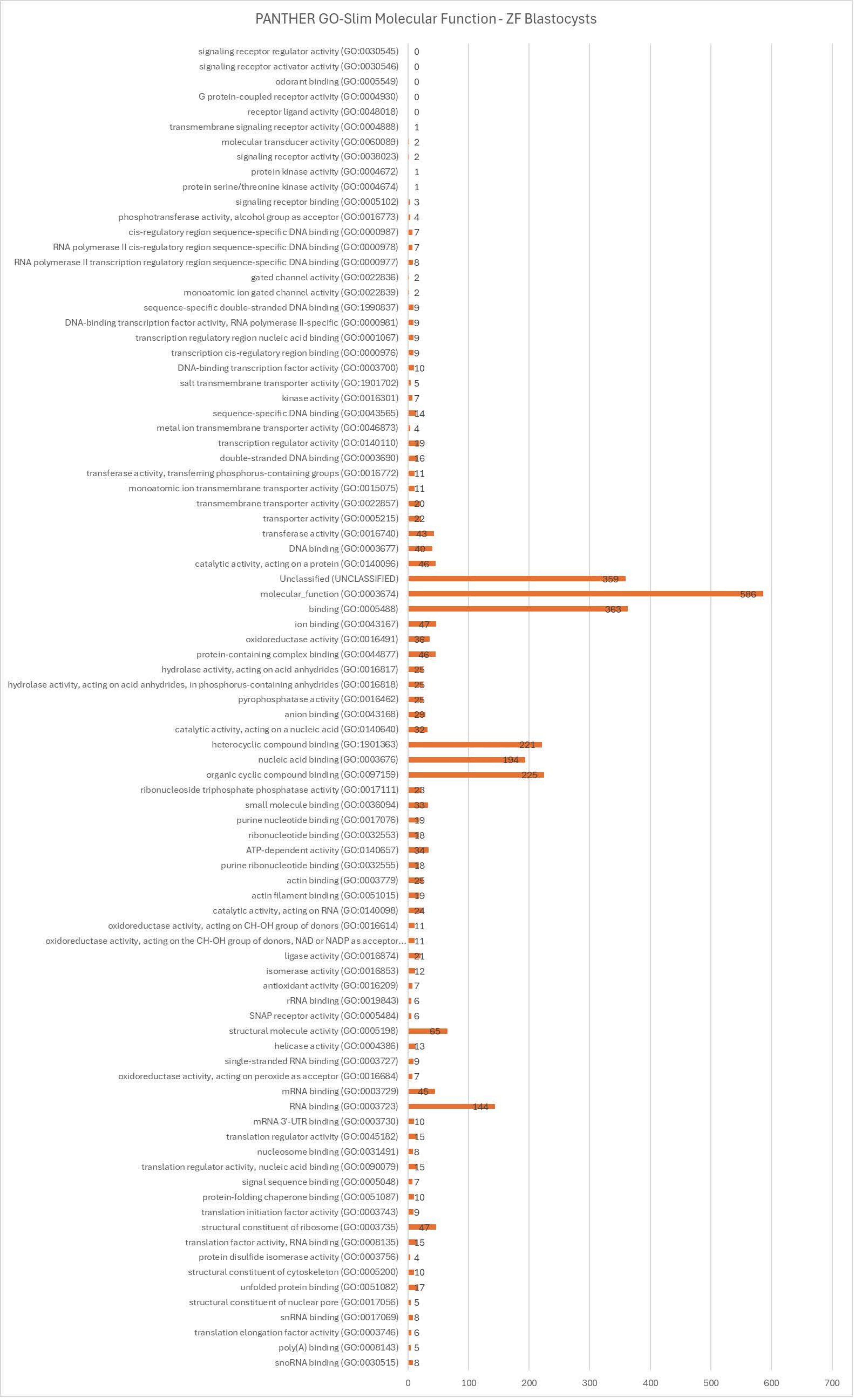
PANTHER GO-Slim Molecular Function - ZF Blastocysts
signaling receptor regulator activity (GO:0030545) 0
signaling receptor activator activity (GO:0030546) 0
odorant binding (GO:0005549) 0
G protein-coupled receptor activity (GO:0004930) 0
receptor ligand activity (GO:0048018) 0
transmembrane signaling receptor activity (GO:0004888) 1
molecular transducer activity (GO:0060089) 2
signaling receptor activity (GO:0038023) 2
protein kinase activity (GO:0004672) 1
protein serine/threonine kinase activity (GO:0004674) 1
signaling receptor binding (GO:0005102) 3
phosphotransferase activity, alcohol group as acceptor (GO:0016773) 4
cis-regulatory region sequence-specific DNA binding (GO:0000987) 7
RNA polymerase II cis-regulatory region sequence-specific DNA binding (GO:0000978) 7
RNA polymerase II transcription regulatory region sequence-specific DNA binding (GO:0000977) 8
gated channel activity (GO:0022836) 2
monoatomic ion gated channel activity (GO:0022839) 2
sequence-specific double-stranded DNA binding (GO:1990837) 9
DNA-binding transcription factor activity, RNA polymerase II-specific (GO:0000981) 9
transcription regulatory region nucleic acid binding (GO:0001067) 9
transcription cis-regulatory region binding (GO:0000976) 9
DNA-binding transcription factor activity (GO:0003700) 10
salt transmembrane transporter activity (GO:1901702) 5
kinase activity (GO:0016301) 7
sequence-specific DNA binding (GO:0043565) 14
metal ion transmembrane transporter activity (GO:0046873) 4
transcription regulator activity (GO:0140110) 19
double-stranded DNA binding (GO:0003690) 16
transferase activity, transferring phosphorus-containing groups (GO:0016772) 11
monoatomic ion transmembrane transporter activity (GO:0015075) 11
transmembrane transporter activity (GO:0022857) 20
transporter activity (GO:0005215) 22
transferase activity (GO:0016740) 43
DNA binding (GO:0003677) 40
catalytic activity, acting on a protein (GO:0140096) 46
Unclassified (UNCLASSIFIED) 359
molecular_function (GO:0003674) 586
binding (GO:0005488) 363
ion binding (GO:0043167) 47
oxidoreductase activity (GO:0016491) 36
protein-containing complex binding (GO:0044877) 46
hydrolase activity, acting on acid anhydrides (GO:0016817) 25
hydrolase activity, acting on acid anhydrides, in phosphorus-containing anhydrides (GO:0016818) 25
pyrophosphatase activity (GO:0016462) 25
anion binding (GO:0043168) 29
catalytic activity, acting on a nucleic acid (GO:0140640) 32
heterocyclic compound binding (GO:1901363) 221
nucleic acid binding (GO:0003676) 194
organic cyclic compound binding (GO:0097159) 225
ribonucleoside triphosphate phosphatase activity (GO:0017111) 23
small molecule binding (GO:0036094) 33
purine nucleotide binding (GO:0017076) 19
ribonucleotide binding (GO:0032553) 18
ATP-dependent activity (GO:0140657) 34
purine ribonucleotide binding (GO:0032555) 18
actin binding (GO:0003779) 25
actin filament binding (GO:0051015) 19
catalytic activity, acting on RNA (GO:0140098) 24
oxidoreductase activity, acting on CH-OH group of donors (GO:0016614) 11
oxidoreductase activity, acting on the CH-OH group of donors, NAD or NADP as acceptor… 11
ligase activity (GO:0016874) 21
isomerase activity (GO:0016853) 12
antioxidant activity (GO:0016209) 7
rRNA binding (GO:0019843) 6
SNAP receptor activity (GO:0005484) 6
structural molecule activity (GO:0005198) 65
helicase activity (GO:0004386) 13
single-stranded RNA binding (GO:0003727) 9
oxidoreductase activity, acting on peroxide as acceptor (GO:0016684) 7
mRNA binding (GO:0003729) 45
RNA binding (GO:0003723) 144
mRNA 3'-UTR binding (GO:0003730) 10
translation regulator activity (GO:0045182) 15
nucleosome binding (GO:0031491) 8
translation regulator activity, nucleic acid binding (GO:0090079) 15
signal sequence binding (GO:0005048) 7
protein-folding chaperone binding (GO:0051087) 10
translation initiation factor activity (GO:0003743) 9
structural constituent of ribosome (GO:0003735) 47
translation factor activity, RNA binding (GO:0008135) 15
protein disulfide isomerase activity (GO:0003756) 4
structural constituent of cytoskeleton (GO:0005200) 10
unfolded protein binding (GO:0051082) 17
structural constituent of nuclear pore (GO:0017056) 5
snRNA binding (GO:0017069) 8
translation elongation factor activity (GO:0003746) 6
poly(A) binding (GO:0008143) 5
snoRNA binding (GO:0030515) 8
0
100
200
300
400
500
600
700

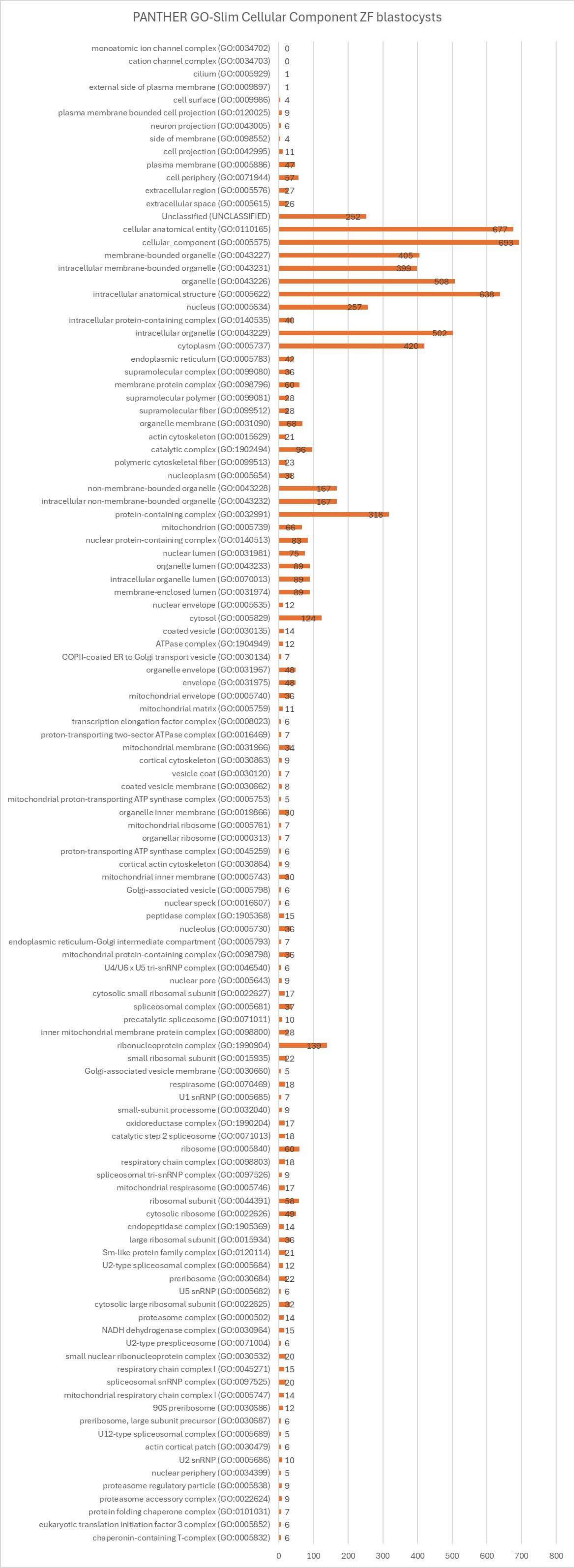
PANTHER GO-Slim Cellular Component ZF blastocysts
monoatomic ion channel complex (GO:0034702) 0
cation channel complex (GO:0034703) 0
cilium (GO:0005929) 1
external side of plasma membrane (GO:0009897) 1
cell surface (GO:0009986) 4
plasma membrane bounded cell projection (GO:0120025) 9
neuron projection (GO:0043005) 6
side of membrane (GO:0098552) 4
cell projection (GO:0042995) 11
plasma membrane (GO:0005886) 47
cell periphery (GO:0071944) 57
extracellular region (GO:0005576) 27
extracellular space (GO:0005615) 26
Unclassified (UNCLASSIFIED) 252
cellular anatomical entity (GO:0110165) 677
cellular_component (GO:0005575) 693
membrane-bounded organelle (GO:0043227) 405
intracellular membrane-bounded organelle (GO:0043231) 399
organelle (GO:0043226) 508
intracellular anatomical structure (GO:0005622) 638
nucleus (GO:0005634) 257
intracellular protein-containing complex (GO:0140535) 40
intracellular organelle (GO:0043229) 502
cytoplasm (GO:0005737) 420
endoplasmic reticulum (GO:0005783) 42
supramolecular complex (GO:0099080) 36
membrane protein complex (GO:0098796) 60
supramolecular polymer (GO:0099081) 28
supramolecular fiber (GO:0099512) 28
organelle membrane (GO:0031090) 68
actin cytoskeleton (GO:0015629) 21
catalytic complex (GO:1902494) 96
polymeric cytoskeletal fiber (GO:0099513) 23
nucleoplasm (GO:0005654) 38
non-membrane-bounded organelle (GO:0043228) 167
intracellular non-membrane-bounded organelle (GO:0043232) 167
protein-containing complex (GO:0032991) 318
mitochondrion (GO:0005739) 66
nuclear protein-containing complex (GO:0140513) 83
nuclear lumen (GO:0031981) 75
organelle lumen (GO:0043233) 89
intracellular organelle lumen (GO:0070013) 89
membrane-enclosed lumen (GO:0031974) 89
nuclear envelope (GO:0005635) 12
cytosol (GO:0005829) 124
coated vesicle (GO:0030135) 14
ATPase complex (GO:1904949) 12
COPII-coated ER to Golgi transport vesicle (GO:0030134) 7
organelle envelope (GO:0031967) 48
envelope (GO:0031975) 48
mitochondrial envelope (GO:0005740) 36
mitochondrial matrix (GO:0005759) 11
transcription elongation factor complex (GO:0008023) 6
proton-transporting two-sector ATPase complex (GO:0016469) 7
mitochondrial membrane (GO:0031966) 34
cortical cytoskeleton (GO:0030863) 9
vesicle coat (GO:0030120) 7
coated vesicle membrane (GO:0030662) 8
mitochondrial proton-transporting ATP synthase complex (GO:0005753) 5
organelle inner membrane (GO:0019866) 30
mitochondrial ribosome (GO:0005761) 7
organellar ribosome (GO:0000313) 7
proton-transporting ATP synthase complex (GO:0045259) 6
cortical actin cytoskeleton (GO:0030864) 9
mitochondrial inner membrane (GO:0005743) 30
Golgi-associated vesicle (GO:0005798) 6
nuclear speck (GO:0016607) 6
peptidase complex (GO:1905368) 15
nucleolus (GO:0005730) 36
endoplasmic reticulum-Golgi intermediate compartment (GO:0005793) 7
mitochondrial protein-containing complex (GO:0098798) 36
U4/U6 x U5 tri-snRNP complex (GO:0046540) 6
nuclear pore (GO:0005643) 9
cytosolic small ribosomal subunit (GO:0022627) 17
spliceosomal complex (GO:0005681) 37
precatalytic spliceosome (GO:0071011) 10
inner mitochondrial membrane protein complex (GO:0098800) 28
ribonucleoprotein complex (GO:1990904) 139
small ribosomal subunit (GO:0015935) 22
Golgi-associated vesicle membrane (GO:0030660) 5
respirasome (GO:0070469) 18
U1 snRNP (GO:0005685) 7
small-subunit processome (GO:0032040) 9
oxidoreductase complex (GO:1990204) 17
catalytic step 2 spliceosome (GO:0071013) 18
ribosome (GO:0005840) 60
respiratory chain complex (GO:0098803) 18
spliceosomal tri-snRNP complex (GO:0097526) 9
mitochondrial respirasome (GO:0005746) 17
ribosomal subunit (GO:0044391) 58
cytosolic ribosome (GO:0022626) 49
endopeptidase complex (GO:1905369) 14
large ribosomal subunit (GO:0015934) 36
Sm-like protein family complex (GO:0120114) 21
U2-type spliceosomal complex (GO:0005684) 12
preribosome (GO:0030684) 22
U5 snRNP (GO:0005682) 6
cytosolic large ribosomal subunit (GO:0022625) 32
proteasome complex (GO:0000502) 14
NADH dehydrogenase complex (GO:0030964) 15
U2-type prespliceosome (GO:0071004) 6
small nuclear ribonucleoprotein complex (GO:0030532) 20
respiratory chain complex I (GO:0045271) 15
spliceosomal snRNP complex (GO:0097525) 20
mitochondrial respiratory chain complex I (GO:0005747) 14
90S preribosome (GO:0030686) 12
preribosome, large subunit precursor (GO:0030687) 6
U12-type spliceosomal complex (GO:0005689) 5
actin cortical patch (GO:0030479) 6
U2 snRNP (GO:0005686) 10
nuclear periphery (GO:0034399) 5
proteasome regulatory particle (GO:0005838) 9
proteasome accessory complex (GO:0022624) 9
protein folding chaperone complex (GO:0101031) 7
eukaryotic translation initiation factor 3 complex (GO:0005852) 6
chaperonin-containing T-complex (GO:0005832) 6
0 100 200 300 400 500 600 700 800

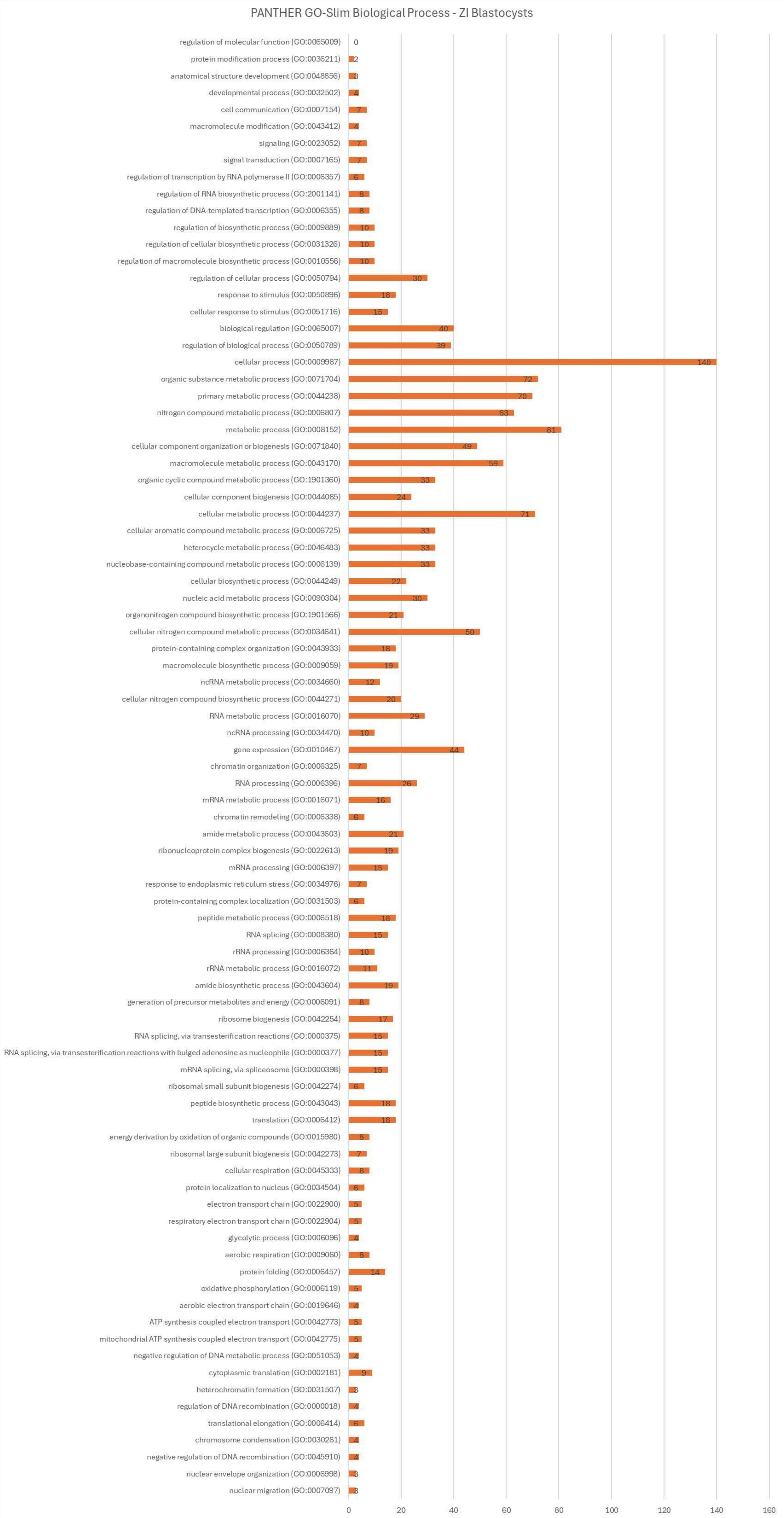
PANTHER GO-Slim Biological Process - ZI Blastocysts
regulation of molecular function (GO:0065009) 0
protein modification process (GO:0036211) 2
anatomical structure development (GO:0048856) 3
developmental process (GO:0032502) 4
cell communication (GO:0007154) 7
macromolecule modification (GO:0043412) 4
signaling (GO:0023052) 7
signal transduction (GO:0007165) 7
regulation of transcription by RNA polymerase II (GO:0006357) 6
regulation of RNA biosynthetic process (GO:2001141) 8
regulation of DNA-templated transcription (GO:0006355) 8
regulation of biosynthetic process (GO:0009889) 10
regulation of cellular biosynthetic process (GO:0031326) 10
regulation of macromolecule biosynthetic process (GO:0010556) 10
regulation of cellular process (GO:0050794) 30
response to stimulus (GO:0050896) 18
cellular response to stimulus (GO:0051716) 15
biological regulation (GO:0065007) 40
regulation of biological process (GO:0050789) 39
cellular process (GO:0009987) 140
organic substance metabolic process (GO:0071704) 72
primary metabolic process (GO:0044238) 70
nitrogen compound metabolic process (GO:0006807) 63
metabolic process (GO:0008152) 81
cellular component organization or biogenesis (GO:0071840) 49
macromolecule metabolic process (GO:0043170) 59
organic cyclic compound metabolic process (GO:1901360) 33
cellular component biogenesis (GO:0044085) 24
cellular metabolic process (GO:0044237) 71
cellular aromatic compound metabolic process (GO:0006725) 33
heterocycle metabolic process (GO:0046483) 33
nucleobase-containing compound metabolic process (GO:0006139) 33
cellular biosynthetic process (GO:0044249) 22
nucleic acid metabolic process (GO:0090304) 30
organonitrogen compound biosynthetic process (GO:1901566) 21
cellular nitrogen compound metabolic process (GO:0034641) 50
protein-containing complex organization (GO:0043933) 18
macromolecule biosynthetic process (GO:0009059) 19
ncRNA metabolic process (GO:0034660) 12
cellular nitrogen compound biosynthetic process (GO:0044271) 20
RNA metabolic process (GO:0016070) 29
ncRNA processing (GO:0034470) 10
gene expression (GO:0010467) 44
chromatin organization (GO:0006325) 7
RNA processing (GO:0006396) 26
mRNA metabolic process (GO:0016071) 16
chromatin remodeling (GO:0006338) 6
amide metabolic process (GO:0043603) 21
ribonucleoprotein complex biogenesis (GO:0022613) 19
mRNA processing (GO:0006397) 15
response to endoplasmic reticulum stress (GO:0034976) 7
protein-containing complex localization (GO:0031503) 6
peptide metabolic process (GO:0006518) 18
RNA splicing (GO:0008380) 15
rRNA processing (GO:0006364) 10
rRNA metabolic process (GO:0016072) 11
amide biosynthetic process (GO:0043604) 19
generation of precursor metabolites and energy (GO:0006091) 8
ribosome biogenesis (GO:0042254) 17
RNA splicing, via transesterification reactions (GO:0000375) 15
RNA splicing, via transesterification reactions with bulged adenosine as nucleophile (GO:0000377) 15
mRNA splicing, via spliceosome (GO:0000398) 15
ribosomal small subunit biogenesis (GO:0042274) 6
peptide biosynthetic process (GO:0043043) 18
translation (GO:0006412) 18
energy derivation by oxidation of organic compounds (GO:0015980) 8
ribosomal large subunit biogenesis (GO:0042273) 7
cellular respiration (GO:0045333) 8
protein localization to nucleus (GO:0034504) 6
electron transport chain (GO:0022900) 5
respiratory electron transport chain (GO:0022904) 5
glycolytic process (GO:0006096) 4
aerobic respiration (GO:0009060) 8
protein folding (GO:0006457) 14
oxidative phosphorylation (GO:0006119) 5
aerobic electron transport chain (GO:0019646) 4
ATP synthesis coupled electron transport (GO:0042773) 5
mitochondrial ATP synthesis coupled electron transport (GO:0042775) 5
negative regulation of DNA metabolic process (GO:0051053) 4
cytoplasmic translation (GO:0002181) 9
heterochromatin formation (GO:0031507) 3
regulation of DNA recombination (GO:0000018) 4
translational elongation (GO:0006414) 6
chromosome condensation (GO:0030261) 4
negative regulation of DNA recombination (GO:0045910) 4
nuclear envelope organization (GO:0006998) 3
nuclear migration (GO:0007097) 3
0
20
40
60
80
100
120
140
160

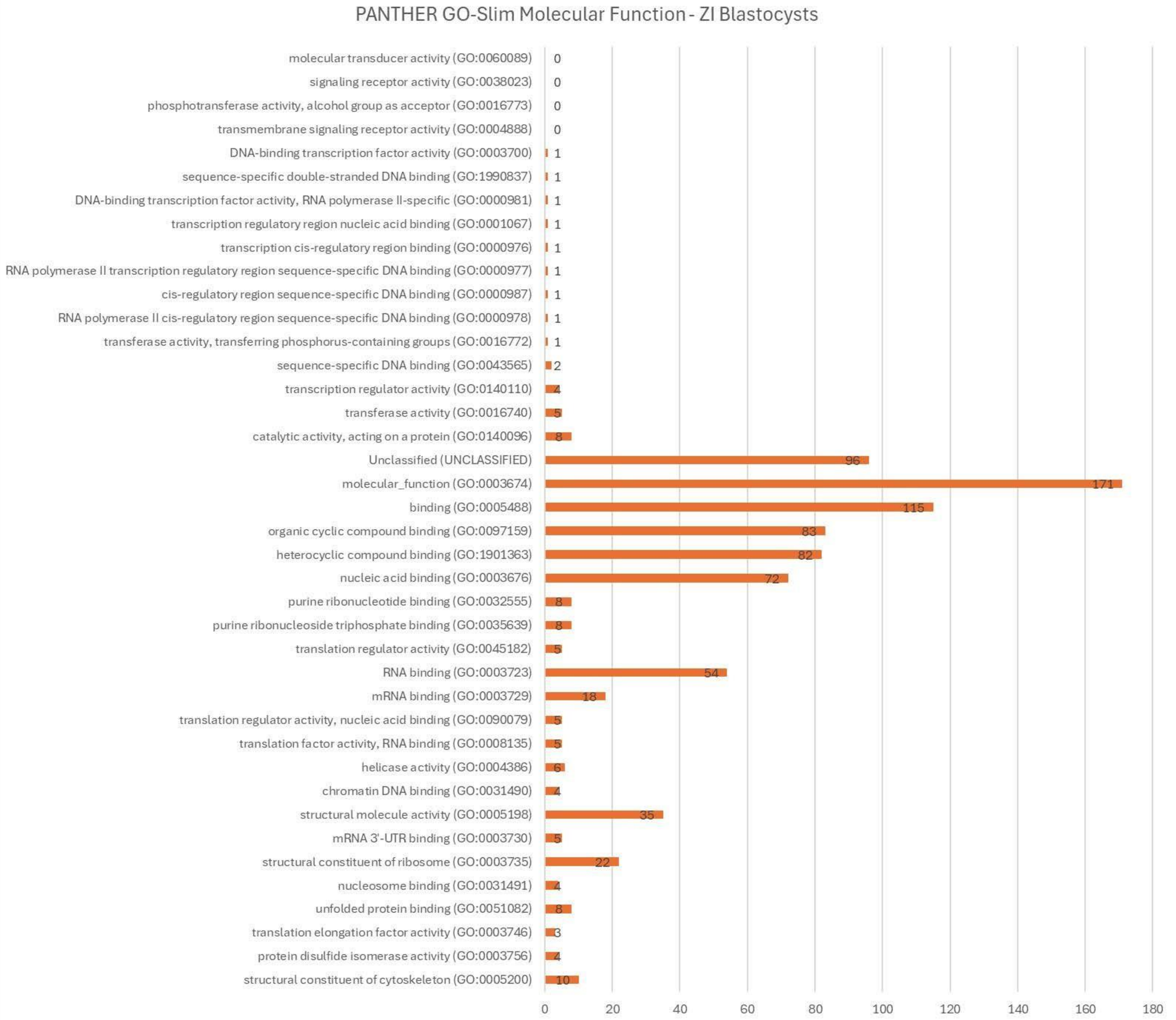

PANTHER GO-Slim Molecular Function - ZI Blastocysts
molecular transducer activity (GO:0060089) 0
signaling receptor activity (GO:0038023) 0
phosphotransferase activity, alcohol group as acceptor (GO:0016773) 0
transmembrane signaling receptor activity (GO:0004888) 0
DNA-binding transcription factor activity (GO:0003700) 1
sequence-specific double-stranded DNA binding (GO:1990837) 1
DNA-binding transcription factor activity, RNA polymerase II-specific (GO:0000981) 1
transcription regulatory region nucleic acid binding (GO:0001067) 1
transcription cis-regulatory region binding (GO:0000976) 1
RNA polymerase II transcription regulatory region sequence-specific DNA binding (GO:0000977) 1
cis-regulatory region sequence-specific DNA binding (GO:0000987) 1
RNA polymerase II cis-regulatory region sequence-specific DNA binding (GO:0000978) 1
transferase activity, transferring phosphorus-containing groups (GO:0016772) 1
sequence-specific DNA binding (GO:0043565) 2
transcription regulator activity (GO:0140110) 4
transferase activity (GO:0016740) 5
catalytic activity, acting on a protein (GO:0140096) 8
Unclassified (UNCLASSIFIED) 96
molecular_function (GO:0003674) 171
binding (GO:0005488) 115
organic cyclic compound binding (GO:0097159) 83
heterocyclic compound binding (GO:1901363) 82
nucleic acid binding (GO:0003676) 72
purine ribonucleotide binding (GO:0032555) 8
purine ribonucleoside triphosphate binding (GO:0035639) 8
translation regulator activity (GO:0045182) 5
RNA binding (GO:0003723) 54
mRNA binding (GO:0003729) 18
translation regulator activity, nucleic acid binding (GO:0090079) 5
translation factor activity, RNA binding (GO:0008135) 5
helicase activity (GO:0004386) 6
chromatin DNA binding (GO:0031490) 4
structural molecule activity (GO:0005198) 35
mRNA 3'-UTR binding (GO:0003730) 5
structural constituent of ribosome (GO:0003735) 22
nucleosome binding (GO:0031491) 4
unfolded protein binding (GO:0051082) 8
translation elongation factor activity (GO:0003746) 3
protein disulfide isomerase activity (GO:0003756) 4
structural constituent of cytoskeleton (GO:0005200) 10
0 20 40 60 80 100 120 140 160 180

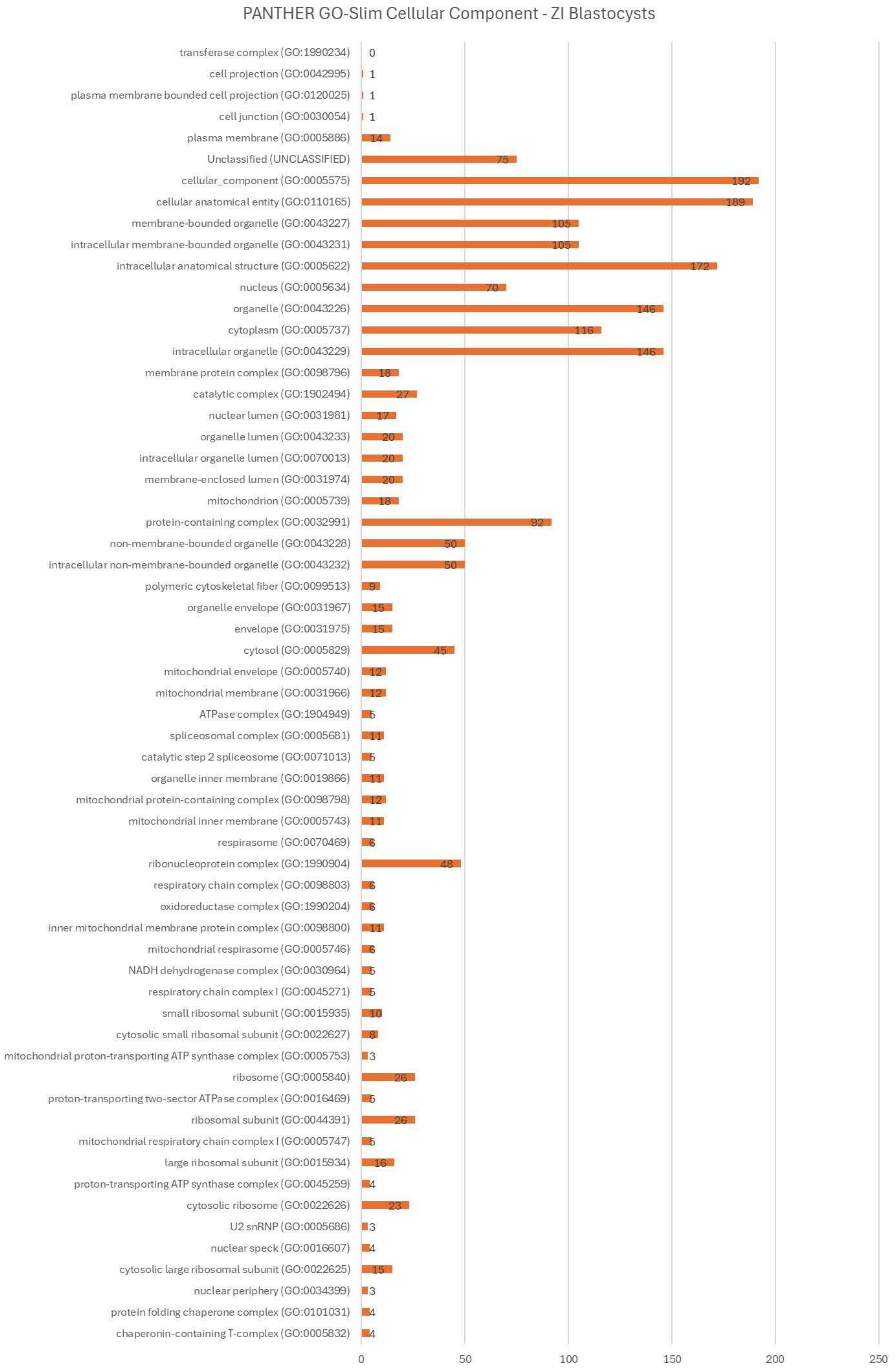
PANTHER GO-Slim Cellular Component - ZI Blastocysts
transferase complex (GO:1990234) 0
cell projection (GO:0042995) 1
plasma membrane bounded cell projection (GO:0120025) 1
cell junction (GO:0030054) 1
plasma membrane (GO:0005886) 14
Unclassified (UNCLASSIFIED) 75
cellular_component (GO:0005575) 192
cellular anatomical entity (GO:0110165) 189
membrane-bounded organelle (GO:0043227) 105
intracellular membrane-bounded organelle (GO:0043231) 105
intracellular anatomical structure (GO:0005622) 172
nucleus (GO:0005634) 70
organelle (GO:0043226) 146
cytoplasm (GO:0005737) 116
intracellular organelle (GO:0043229) 146
membrane protein complex (GO:0098796) 18
catalytic complex (GO:1902494) 27
nuclear lumen (GO:0031981) 17
organelle lumen (GO:0043233) 20
intracellular organelle lumen (GO:0070013) 20
membrane-enclosed lumen (GO:0031974) 20
mitochondrion (GO:0005739) 18
protein-containing complex (GO:0032991) 92
non-membrane-bounded organelle (GO:0043228) 50
intracellular non-membrane-bounded organelle (GO:0043232) 50
polymeric cytoskeletal fiber (GO:0099513) 9
organelle envelope (GO:0031967) 15
envelope (GO:0031975) 15
cytosol (GO:0005829) 45
mitochondrial envelope (GO:0005740) 12
mitochondrial membrane (GO:0031966) 12
ATPase complex (GO:1904949) 5
spliceosomal complex (GO:0005681) 11
catalytic step 2 spliceosome (GO:0071013) 5
organelle inner membrane (GO:0019866) 11
mitochondrial protein-containing complex (GO:0098798) 12
mitochondrial inner membrane (GO:0005743) 11
respirasome (GO:0070469) 6
ribonucleoprotein complex (GO:1990904) 48
respiratory chain complex (GO:0098803) 6
oxidoreductase complex (GO:1990204) 6
inner mitochondrial membrane protein complex (GO:0098800) 11
mitochondrial respirasome (GO:0005746) 6
NADH dehydrogenase complex (GO:0030964) 5
respiratory chain complex I (GO:0045271) 5
small ribosomal subunit (GO:0015935) 10
cytosolic small ribosomal subunit (GO:0022627) 8
mitochondrial proton-transporting ATP synthase complex (GO:0005753) 3
ribosome (GO:0005840) 26
proton-transporting two-sector ATPase complex (GO:0016469) 5
ribosomal subunit (GO:0044391) 26
mitochondrial respiratory chain complex I (GO:0005747) 5
large ribosomal subunit (GO:0015934) 16
proton-transporting ATP synthase complex (GO:0045259) 4
cytosolic ribosome (GO:0022626) 23
U2 snRNP (GO:0005686) 3
nuclear speck (GO:0016607) 4
cytosolic large ribosomal subunit (GO:0022625) 15
nuclear periphery (GO:0034399) 3
protein folding chaperone complex (GO:0101031) 4
chaperonin-containing T-complex (GO:0005832) 4
0
50
100
150
200
250

Table **S4. Proteins identified in the culture medium of blastocysts from the ZF and ZI groups**.

	Protein ID	Description	Name	Group
1	A0A5F5XYD4_FELCA	Keratin, type I cytoskeletal	KRT15	ZF and ZI
2	M3VXR1_FELCA	Keratin 10	KRT10	ZF and ZI
3	M3X1M2_FELCA	Hemicentin 1	HMCN1	ZF and ZI
4	P49064_FELCA	Albumin	ALB	ZF and ZI
5	A0A337S8S6_FELCA	Protease, Serine, 2 (Trypsin 2)	TRY2	ZF and ZI
6	A0A337SCP9_FELCA	Keratin, type I cytoskeletal 13	KRT13	ZF and ZI
7	M3VXK4_FELCA	Keratin, type I cytoskeletal 17	KRT17	ZF and ZI
8	M3VUG7_FELCA	Keratin 76	KRT76	ZF and ZI
9	A0A5F5XLA0_FELCA	Keratin, type I cytoskeletal 17	KRT17	ZF and ZI
10	M3VUF9_FELCA	Keratin 74	KRT74	ZF and ZI
11	M3VUG8_FELCA	Keratin 3	KRT3	ZF and ZI
12	M3VUG4_FELCA	Intermediate filament protein	unassigned	ZF and ZI
13	A0A2I2U8D2_FELCA	Centrosomal protein of 162 kDa	CEP162	ZF and ZI
14	A0A337SFR9_FELCA	GDNF family receptor alpha 3	GFRA3	ZF and ZI
15	A0A5F5XDM4_FELCA	Actin beta	ACTB	ZF and ZI
16	A0A5F5XNI1_FELCA	Transferrin	TF	ZF and ZI
17	M3WLU0_FELCA	Cathepsin B	CTSB	ZF and ZI
18	A0A5F5XLU4_FELCA	Pantetheinase	VNN1	ZF and ZI
19	A0A2I2UZM2_FELCA	Heat shock protein family A (Hsp70) member 8	HSPA8	ZF and ZI
20	M3X9A1_FELCA	Triosephosphate isomerase	TPI1	ZF and ZI
21	M3VUU7_FELCA	Calreticulin	CALR	ZF and ZI
22	M3WZD7_FELCA	Belongs to the intermediate filament family	KRT89	ZF and ZI
23	A0A5F5XZ97_FELCA	Fatty acid-binding protein 5	FABP5	ZF and ZI
24	A0A337SJA7_FELCA	Calmodulin 2	CALM2	ZF and ZI
25	A0A337S1R3_FELCA	Prothymosin alpha	PTMA	ZF and ZI
1	A0A337SNF5_FELCA	Alpha-ketoglutarate-dependent dioxygenase	FT5	ZF
2	A0A337S839_FELCA	Protein Mdm4	MDM4	ZF
3	A0A337S8L1_FELCA	L-lactate dehydrogenase	LDHA	ZF
4	A0A337S103_FELCA	Metallothionein	MT1X	ZF
5	M3W8Q7_FELCA	Glutamate dehydrogenase [NAD(P)(+)]	GLUD1	ZF
1	A0A2I2USF3_FELCA	Histone H2B	H2BC17	ZI
2	A0A5F5XCD9_FELCA	Belongs to the intermediate filament family	KRT86	ZI
3	A0A337SCD2_FELCA	Annexin	ANXA2	ZI
4	A0A2I2UBN8_FELCA	Junction plakoglobin	JUP	ZI
5	M3W114_FELCA	Proteolipid protein 2	PLP2	ZI
6	A0A2I2UGZ3_FELCA	Keratin 32	KRT32	ZI
7	A0A5F5XSP5_FELCA	Leucine aminopeptidase 3	LAP3	ZI
8	M3W2I5_FELCA	Plakophilin 1	PKP1	ZI
9	M3WCK3_FELCA	Desmoplakin	DSP	ZI
10	M3VUF5_FELCA	Keratin 82	KRT82	ZI
11	M3W584_FELCA	H1.5 linker histone, cluster member	H1-5	ZI
12	A0A5K1VP01_FELCA	Lamin A/C	LMNA	ZI

13	A0A337SS59_FELCA	Sad1 and UNC84 domain containing 2	SUN2	ZI
14	A0A2I2U7V8_FELCA	Four and a half LIM domains 1	FHL1	ZI

Figure S5. GO annotation of the proteins identified in the culture medium of ZF and ZI blastocysts.

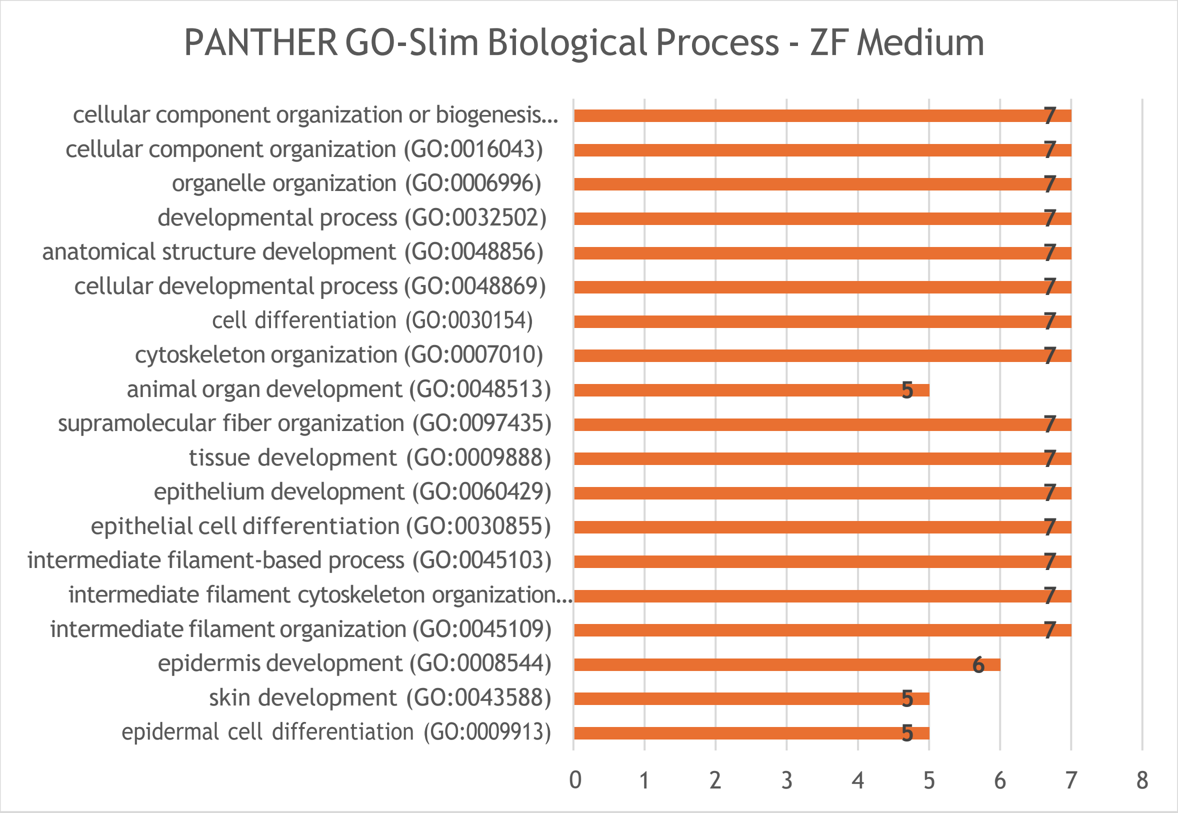

S5.1. GO Biological Process annotation of all the proteins identified in the medium samples of ZF Blastocysts. The PANTHER18.0 Classification System was used for analysis.

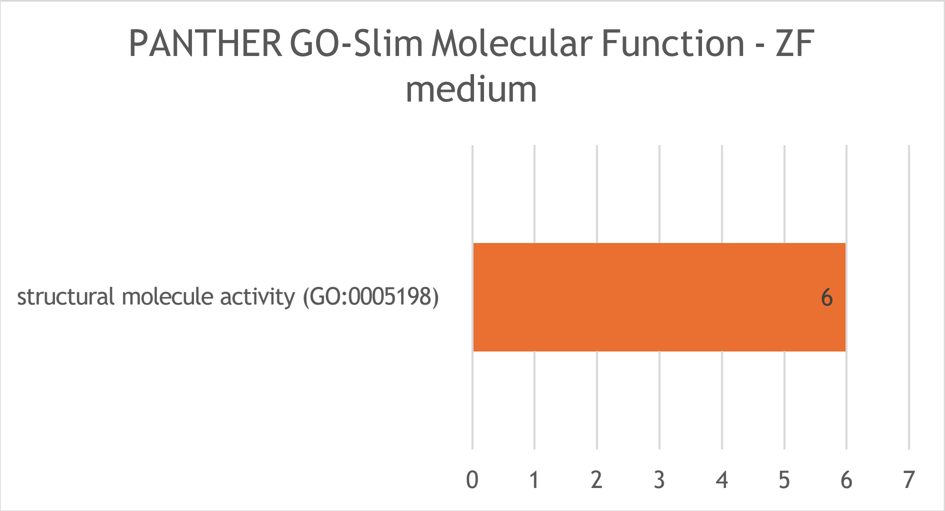

S5.2. GO Molecular Function annotation of all the proteins identified in the medium samples of ZF Blastocysts. The PANTHER18.0 Classification System was used for analysis.

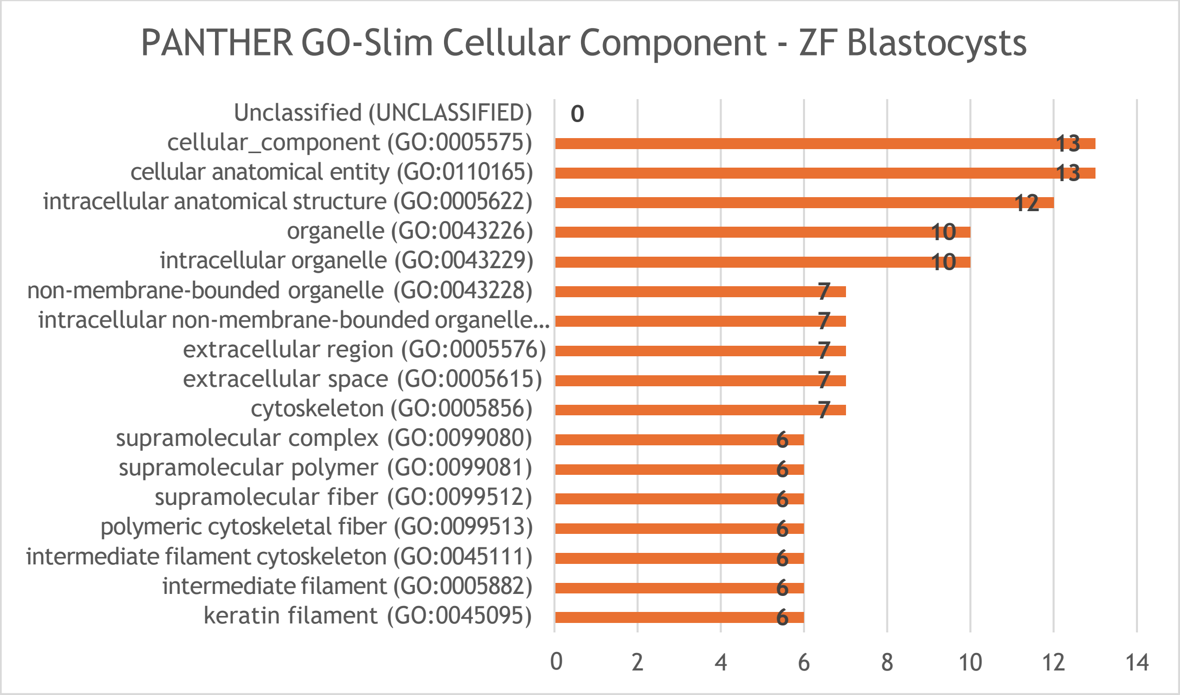

S5.3. GO Cellular Component annotation of all the proteins identified in the medium samples of ZF Blastocysts. The PANTHER18.0 Classification System was used for analysis.

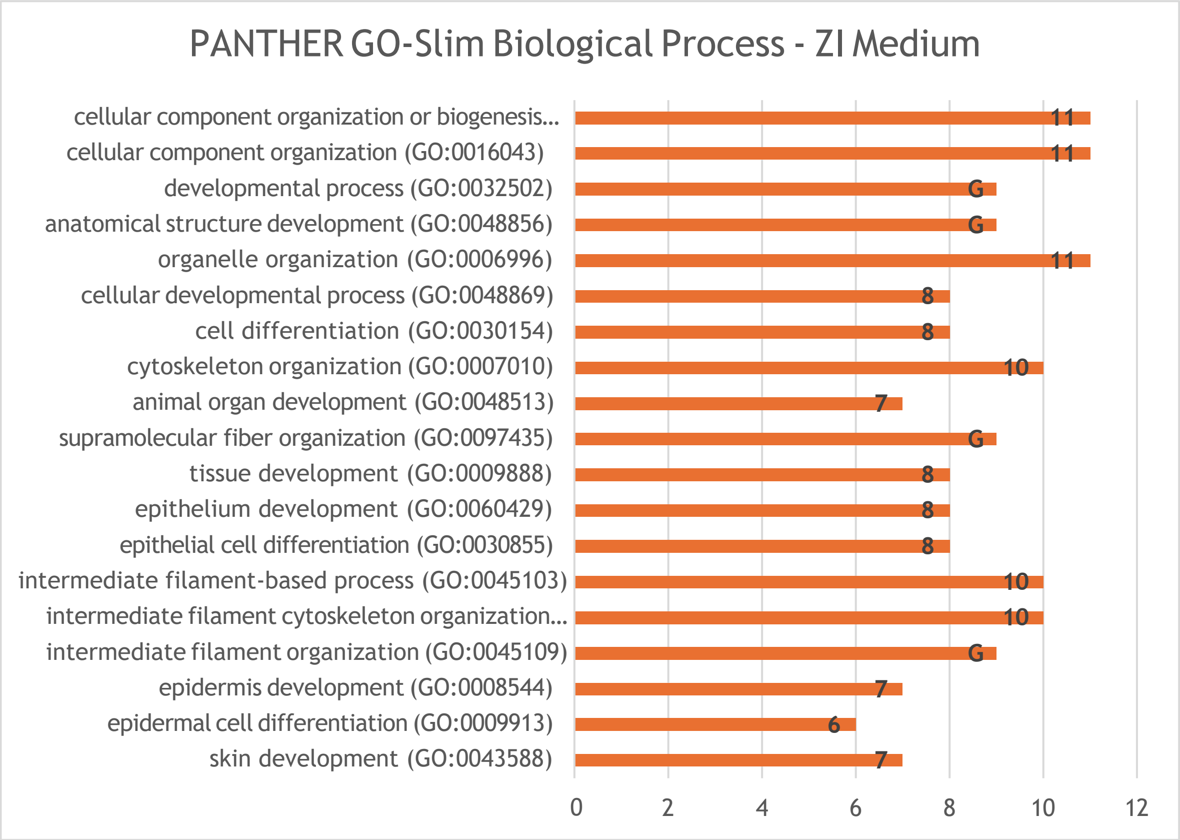

S5.4. GO Biological Process annotation of all the proteins identified in the medium samples of ZI Blastocysts. The PANTHER18.0 Classification System was used for analysis.

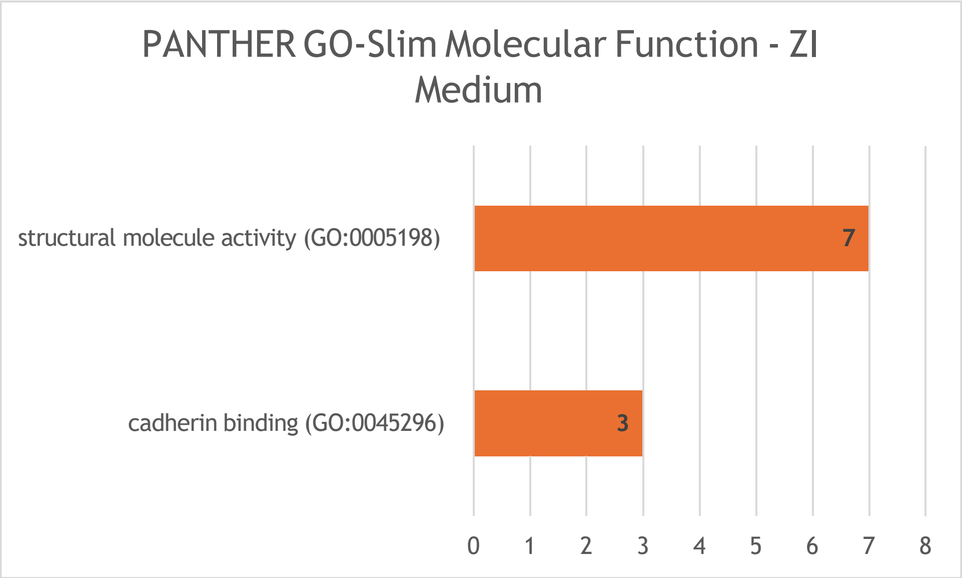

S5.5. GO Molecular Function annotation of all the proteins identified in the medium samples of ZI Blastocysts. The PANTHER18.0 Classification System was used for analysis.

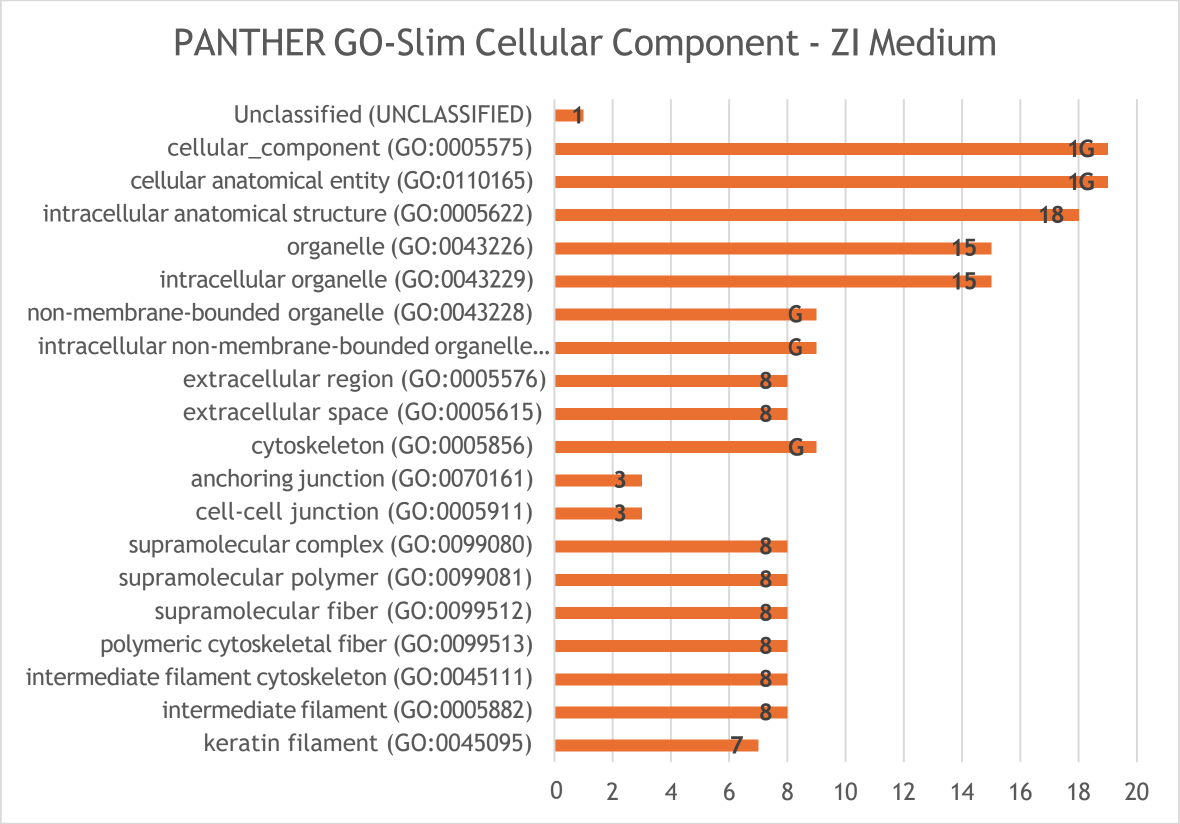

S5.6. GO Cellular Component annotation of all the proteins identified in the medium samples of ZI Blastocysts. The PANTHER18.0 Classification System was used for analysis.

Table S6. Detailed functional annotation of DEPs (Fig.8 Pie charts). Proteins identified in the culture medium of ZF blastocysts (A, B, and C), ZI blastocysts (D, E, and F), and proteins shared between the conditioned medium of ZF and ZI blastocysts (G, H, and I).

A) ZF-medium. Molecular function GO:0003674

1. Binding GO:0005488 (FT5, MDM4, MT1X).	3
2. Catalytic activity GO:0003824 (FT5, LDHA, GLUD1).	3

B) ZF-medium. Biological process GO:0008150

1. Metabolic process GO:0008152 (FT5, LDHA, GLUD1).	3
2. Cellular process GO:0009987 (FT5, MDM4, LDHA, GLUD1).	4
3. Response to stimulus GO:0050896 (FT5).	1
4. Biological regulation GO:0065007 (FT5, MDM4).	2
5. Developmental process GO:0032502 (FT5).	1
6. Growth GO:004007 (FT5).	1
7. Localization GO:0051179 (GLUD1).	1
8. Unassigned (MT1X).	1

C) ZF-medium. Cellular component GO:0005575

1. Cytoplasm GO:0005737 (FT5).	1
2. Nucleoplasm GO:0005654 (FT5).	1
3. Nucleus GO:0005634 (MDM4).	1
4. Mitochondrion GO:0005739 (LDHA, GLUD1).	2
5. Organelle lumen GO:0043233 (GLUD1).	1
6. Unassigned (MT1X).	1

D) ZI-medium. Molecular function GO:0003674

1. Binding GO:0005488 (H2BC17, ANXA2, JUP, PKP1, H1-5, FHL1).	6
2. Structural molecule activity GO:0005198 (KRT86, KRT32, KRT82).	3
3. Molecular function regulator activity G0:0098772 (ANXA2).	1
4. Transporter activity GO:0005198 (ANXA2).	1
5. Transcription regulator activity GO:0140110 (JUP).	1
6. Catalytic activity GO:0003824, (LAP3).	1
7. Unassigned (PLP2, DSP, LMNA, SUN2).	4

E) ZI-medium. Biological process GO:0008150

1. Biological regulation GO:0065007 (JUP, H1-5).	2
2. Cellular process GO:0009987 (JUP, PKP1, DSP, KRT82, H1-5).	5
3. Response to stimulus GO:0050896 (JUP).	1
4. Metabolic process GO:0008152 (LAP3).	1
5. Developmental process GO:0032502 (DSP, KRT82, FHL1).	3
6. Multicellular organismal process GO:0032501 (KRT82).	1
7. Unassigned (H2BC17, KRT86, ANXA2, PLP2, KRT32, LMNA, SUN2).	7

F) ZI-medium. Cellular component GO:0005575

1. Nucleus GO:0005634 (H2BC17, ANXA2, JUP, PKP1, H1-5, LMNA).	6
2. Protein-containing complex GO:0032991 (H2BC17, ANXA2, H1-5).	3
3. non-membrane-bounded organelle GO:0043228 (H2BC17, ANXA2, H1-5).	3
4. Cytoskeleton GO:0005856 (KRT86, JUP, KRT32, KRT82, LMNA, DSP).	6
5. Extracellular region GO:0005576 (ANXA2).	1
6. Endosome GO:0005768 (ANXA2).	1
7. Vacuole GO:0005773 (ANXA2).	1
8. Cytosol GO:0005829 (ANXA2, JUP, LAP3).	3
9. Plasma membrane GO:0005886 (ANXA2, JUP, PKP1, DSP).	4
10. Organelle GO:0043226 (JUP).	1
11. Membrane GO:0016020 (PLP2, SUN2).	2
12. Nucleoplasm GO:0005635 (PKP1).	1
13. Organelle lumen GO:0043233 (H1-5).	1
14. Nuclear envelope GO:0005635 (LMNA).	1
15. Unassigned (FLH1).	1

G) ZF and ZI medium. Molecular function GO:0003674

1. Structural molecule activity GO:0005198 (KRT15, KRT10, KRT13, KRT17, KRT76, KRT74, KRT3, LOC101097497, KRT89).	10
2. Binding GO:0005488 (KRT10, HMCN1, ALB, ACTB, CTSB, HSPA8, TPI1, CALR, FABP5, CALM2, PTMA).	11
3. Catalytic activity GO:0003824 (TRY2, CTSB, VNN1, HSPA8, TPI1).	5
4. Molecular transducer activity GO:0060089 (GFRA3).	1
5. ATP-dependent activity GO:0140657 (HSPA8).	1
6. Protein folding chaperone GO:0044183 (HSPA8).	1
7. Molecular carrier activity GO:0140104 (CALR).	1
8. Unassigned (CEP162, TF).	2

H) ZF and ZI medium. Biological process GO:0008150

1. Cellular process GO:0009987 (KRT10, HMCN1, ALB, KRT13, KRT76, KRT74, KRT3, LOC101097497, CEP162, CTSB, HSPA8, TPI1, CALR, KRT89, PTMA).	15
2. Metabolic process GO:0008152 (TRY2, CTSB, VNN1, HSPA8, TPI1, CALR, PTMA).	7
3. Developmental process GO:0032502 (KRT10, KRT13, KRT76, KRT74, KRT3, LOC101097497, CTSB, CALR, KRT89).	9
4. Response to stimulus GO:0050896 (HMCN1, ALB, KRT13, CTSB, CALR).	5
5. Biological regulation GO:0065007 (ALB, KRT13, KRT74, CALR, PTMA).	5
6. Multicellular organismal process GO:0032501 (KRT74, LOC101097497, CTSB, CALR).	4
7. Reproduction GO:0000003 (CTSB, CALR).	2
8. Localization GO:0051179 (HSPA8, CALR).	2
9. Immune system process GO:0002376 (CALR).	1
10. Unassigned (KRT15, KRT17, GFRA3, ACTB, TF, FABP5, CALM2).	7

I) ZF and ZI medium. Cellular component GO:0005575

1. Cytoskeleton GO:0005856 (KRT15, KRT10, KRT13, KRT17, KRT76, KRT74, KRT3, LOC101097497, CEP162, KRT89).	10
2. Cytoplasm GO:0005737 (KRT10, ALB, KRT74, CEP162, FABP5).	5
3. Plasma membrane GO:0005886 (KRT10, KRT74, GFRA3, CTSB, HSPA8, CALR).	6
4. Extracellular region GO:0005576 (HMCN1, CTSB, FABP5).	3
5. Extracellular matrix GO:0031012 (HMCN1).	1
6. Extracellular space GO:0005615 (ALB, TF, CALR).	3
7. Protein-containing complex GO:0032991 (ALB, CTSB).	2
8. Microtubule organizing center GO:0005815 (CEP162).	1
9. Vacuole GO:0005773 (CTSB, HSPA8).	2
10. Cellular anatomical entity GO:0110165 (VNN1).	1
11. Nucleus GO:0005634 (HSPA8, PTMA).	2
12. Cytosol GO:0005829 (HSPA8, TPI1, CALR, PTMA).	4
13. Cell projection GO:0042995 (HSPA8).	1
14. Nuclear envelope GO:0005635 (CALR).	1
15. Endoplasmic reticulum GO:0005783 (CALR).	1
16. Unassigned (TRY2, ACTB, CALM2).	3
